# Effects of Modified Electroconvulsive Therapy on Serum Cortisol, Nesfatin-1, and Pro-inflammatory Cytokine Levels in Elderly Patients With Treatment-Resistant Depression

**DOI:** 10.3389/fendo.2022.904005

**Published:** 2022-06-16

**Authors:** Biao Dai, Xiaoping Wu, Fanfan Yan, Yang Chen, Yayun Xu, Qingrong Xia, Xulai Zhang, Xuefeng Xie

**Affiliations:** ^1^ Department of Basic and Clinical Pharmacology, Anhui Institute of Innovative Drugs, School of Pharmacy, Anhui Medical University, Hefei, China; ^2^ Affiliated Psychological Hospital of Anhui Medical University, Hefei, China; ^3^ Department of Pharmacy, Hefei Fourth People’s Hospital, Hefei, China; ^4^ Department of Medical Education and Research, Anhui Mental Health Center, Hefei, China; ^5^ Department of Geriatric Psychology, Hefei Fourth People’s Hospital, Hefei, China; ^6^ Modified Electroconvulsive Therapy Room, Hefei Fourth People’s Hospital, Hefei, China; ^7^ Department of Epidemiology and Biostatistics, School of Public Health, Anhui Medical University, Hefei, China; ^8^ Department of Science and Education, Hefei Fourth People’s Hospital, Hefei, China; ^9^ Anhui Provincial Clinical Research Center for Mental and Mental Diseases, Hefei, China

**Keywords:** treatment-resistant depression, Nesfatin-1, Cortisol, pro-inflammatory cytokines, modified electroconvulsive therapy, elderly

## Abstract

**Aim:**

Modified electroconvulsive therapy (MECT) is an effective strategy for treatment-resistant depression (TRD); however, the mechanism underlying effects of MECT remains unclear. Accumulating evidence suggests that TRD is closely associated with dysfunction of the hypothalamic-pituitary-adrenal (HPA) axis, anorexigenic peptides, and pro-inflammatory cytokines. However, MECT effects on the HPA axis, anorexigenic peptides, and pro-inflammatory cytokines in elderly patients with TRD remain unclear. In this study, we investigated whether the HPA axis (cortisol), anorexigenic peptides (nesfatin-1), and pro-inflammatory cytokines (C-reactive protein, tumor necrosis factor-α, and interleukin-6, and interleukin-1β) are involved in the mechanism underlying MECT effects in elderly patients with TRD.

**Methods:**

Elderly patients with TRD were enrolled in this study between December 2019 and October 2021; all patients underwent MECT after physical examination. Serum cortisol, nesfatin-1, and pro-inflammatory cytokine levels were measured before and after the first, third, and sixth MECT sessions. The Hamilton Depression Rating Scale-24 (HAMD-24) and the Mini-Mental State Examination (MMSE) were used to evaluate depression and cognitive impairment, respectively. We compared pre- and post-MECT serum cortisol, nesfatin-1, and pro-inflammatory cytokine levels to confirm the short-term effects of MECT on these serum indices. We compared these serum indices across three time points (before the first, third, and sixth MECT sessions) to determine the long-term effects of MECT on serum cortisol, nesfatin-1, and pro-inflammatory cytokine levels.

**Results:**

We observed no statistically significant changes in the pre- and post-MECT serum cortisol, nesfatin-1, or pro-inflammatory cytokine levels. No significant changes in serum cortisol, nesfatin-1, and pro-inflammatory cytokine levels were observed across the aforementioned time points. Moreover, there were no statistically significant sex-based differences in the aforementioned serum indices. Furthermore, the serum cortisol level was negatively correlated with the serum IL-6 level before and after the first MECT session in patients with high cortisol levels (> the 50^th^ percentile value of all samples). Additionally, the post-MECT HAMD-24 and MMSE scores were significantly lower.

**Conclusions:**

MECT reduced depressive symptoms despite an adverse effect on cognition and had no significant effect on the serum cortisol, nesfatin-1, and pro-inflammatory cytokine levels in elderly patients with TRD.

## Introduction

Depression, one of the most common mental disorders, is a leading cause of disability and is associated with a significant socioeconomic burden worldwide. A recent large-scale survey in China showed that the lifetime prevalence of depressive disorders was 6.9% (1). Although antidepressants serve as first-line treatment for major depressive disorder (MDD), approximately 30% of patients with MDD, particularly elderly patients, fail to respond to antidepressant therapy, leading to treatment-resistant depression (TRD) (2, 3). Modified electroconvulsive therapy (MECT) is a safe and effective therapeutic modality for patients with MDD and TRD. It has been reported that the effective rate of MECT for MDD was 80–90% (4, 5). A meta-analysis reported that MECT is perhaps more effective than drug therapy (5). However, the exact mechanism that contributes to the effectiveness of MECT in the management of TRD remains unknown.

Accumulating evidence suggests a close association between hypothalamic-pituitary-adrenal (HPA) axis disruption and TRD. Dysregulation of the HPA axis, particularly glucocorticoid resistance (which leads to chronically elevated cortisol levels), commonly occurs in patients with TRD and appears to play a key role in its pathophysiology (6). Moreover, chronically elevated cortisol levels are associated with poorer outcomes inpatients treated for MDD, which results in ‘treatment-resistance’ in some patients (7). In this study, we investigated the effects of MECT on serum cortisol levels in elderly patients with TRD.

Recent studies have implicated anorexigenic or orexigenic peptides in the pathogenesis of depression (8, 9). Nesfatin-1, a newly discovered anorexigenic peptide, was shown to be upregulated in peripheral blood and was positively correlated with the severity of depression (10, 11). A recent clinical study showed that blood nesfatin-1 levels were slightly higher in patients with manic episodes treated with electroconvulsive therapy (ECT) and antipsychotic treatment than in patients who received antipsychotic monotherapy (12), which suggests that nesfatin-1 may play a role in ECT. Therefore, we investigated whether MECT affected serum nesfatin-1 levels in elderly patients with TRD.

Immune dysregulation is increasingly being recognized as a potentially major contributor to the pathophysiology of TRD (13, 14). Patients with MDD who show increased levels of serum pro-inflammatory cytokines, including C-reactive protein (CRP), tumor necrosis factor-α (TNF-α), interleukin-6 (IL-6), and IL-1β, had a poor response to conventional antidepressant therapies (15–18). A meta-analysis reported that ECT initially resulted in increased serum IL-6 levels and potentially decreased TNF-α levels in patients with TRD (13). Therefore, it is reasonable to conclude that MECT may affect pro-inflammatory cytokine levels in elderly patients with TRD.

In view of the role of dysfunction of the HPA axis, anorexigenic peptides, and pro-inflammatory cytokines in the pathophysiology of TRD and the potential association between MECT and these serum indices, we investigated whether the HPA axis (cortisol), anorexigenic peptides (nesfatin-1), and pro-inflammatory cytokines (CRP, TNF-α, IL-6, and IL-1β) play a role in the mechanism that contributes to the effects of MECT in elderly patients with TRD.

## Materials and Methods

### Subjects

This study was performed at the Department of Geriatric Psychology, Hefei Fourth People’s Hospital, Anhui Mental Health Center between December 2019 and October 2021. Elderly patients with TRD were screened by an experienced researcher using psychiatric interviews in accordance with the guidelines of the structured clinical interview based on the Diagnostic and Statistical Manual of Mental Disorders-Fourth Edition (DSM-IV). Inclusion criteria were as follows: (1) age ≥ 60 years, (2) meeting the diagnostic criteria of depression listed in the DSM-IV, (3) Hamilton Depression Rating Scale-24 (HAMD-24) scores > 21 and, (4) unresponsiveness to optimal doses of ≥ two antidepressants of different chemical structures administered over 3 months. Exclusion criteria were as follows: (1) A history of other major psychiatric disorders or neurodegenerative illnesses, (2) substance abuse (drug, caffeine, nicotine, alcohol, or others), (3) serious heart, brain, liver, kidney, immune disorders, and/or obesity, poor nutrition, acute and chronic infection and, (4) ECT administration. We included 30 elderly patients with TRD (19 women and 11 men). Patients’ mean age was 66.40 ± 1.39 years, and the mean body mass index was 23.03 ± 0.70 kg/m^2^. The study protocol was approved by the Ethics Committee of the Anhui Mental Health Center, and informed consent was obtained from all patients.

### Modified Electroconvulsive Therapy Procedure

All MECT procedures were performed based on previously described methods with slight modifications (19, 20). Briefly, patients underwent the MECT between 8:00 and 11:00 a.m. using a Thymatron System IV Integrated ECT System (Somatics, Lake Bluff, IL) at the Anhui Mental Health Center. Patients underwent 6 MECT sessions (twice a week for 3 weeks). The initial percentage energy dial setting was based on the patient’s age (for example, 63% for a 63-year-old patient). If no seizure activity was observed, the percent energy increased until a therapeutically satisfactory seizure was observed. Patients received propofol anesthesia during each ECT procedure. Succinylcholine and atropine were administered for muscle relaxation and suppression of glandular secretions, respectively, and electroencephalography was performed to monitor seizure activity.

### Measurements

Blood samples were obtained before and after the first, third, and sixth MECT sessions (before: approximately at 7:50 a.m. and after: approximately at 11:10 a.m.). Samples were immediately centrifuged at 1, 200 g at 4°C for 10 min. The supernatant was extracted as a serum sample, which was stored at -80°C until detection. Commercially available enzyme-linked immunosorbent assay kits were used to measure serum levels of cortisol (catalog number: JL12377), nesfatin-1 (catalog number: JL19919), CRP (catalog number: JL13865), IL-6 (catalog number: JL14113), IL-1β (catalog number: JL19263), and TNF-α (catalog number: JL10208) (Jianglai Bio, Shanghai, China) based on the manufacturer’s instructions.

### Statistical Analysis

All data were analyzed using the SPSS software, version 17.0 (IBM Corp., Armonk, NY, USA). Data are expressed as mean ± standard error of the mean, and *P* < 0.05 was considered statistically significant. A one-sample Kolmogorov-Smirnov test was used for analysis of normally distributed continuous variables. A paired-samples t-test was used to compare the pre- and post-MECT data. The chi-squared test was used for dichotomous variables. Intergroup differences based on the increase and decrease in serum indices before and after the sixth treatment were compared using the Student’s t-test. Correlation analysis was performed using the Pearson’s test. One-way repeated measures analysis of variance (ANOVA) and the Bonferroni correction as a *post hoc* test were used to compare the long-term effects of MECT on the HAMD-24 and MMSE scores, serum cortisol, nesfatin-1, and pro-inflammatory cytokine levels across three time points.

## Results

### Short-Term Effects of Modified Electroconvulsive Therapy on Serum Cortisol, Nesfatin-1, and Pro-Inflammatory Cytokine Levels

**Figure 1:**
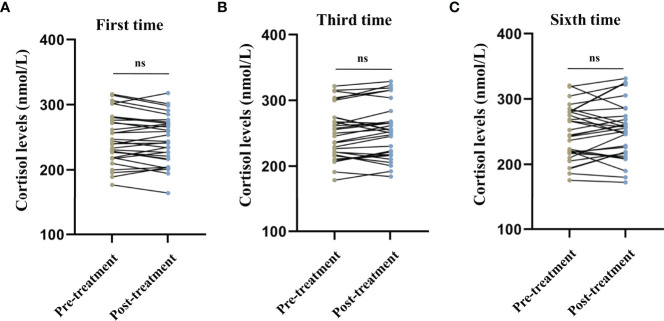
Comparison of serum cortisol levels before and after modified electroconvulsive therapy. **(A)** Comparison of serum cortisol levels before and after the first MECT session. **(B)** Comparison of serum cortisol levels before and after the third MECT session. **(C)** Comparison of serum cortisol levels before and after the sixth MECT session. MECT, modified electroconvulsive therapy; ns, no significance.

**Figure 2:**
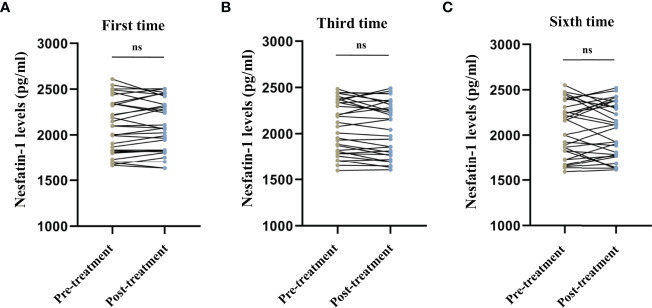
Comparison of serum nesfatin-1 levels before and after modified electroconvulsive therapy. **(A)** Comparison of serum nesfatin-1 levels before and after the first MECT session. **(B)** Comparison of serum nesfatin-1 levels before and after the third MECT session. **(C)** Comparison of serum nesfatin-1 levels before and after the sixth MECT session. MECT, modified electroconvulsive therapy; ns, no significance.

No significant differences were observed in the serum cortisol levels before and after the first (*t* = 1.243, *P* = 0.224, **Figure 1A**), third (*t* = -1.673, *P* = 0.105, **Figure 1B**), and sixth (*t* = -1.124, *P* = 0.270, **Figure 1C**) MECT sessions in elderly patients with TRD. Similarly, no statistical difference was observed in serum nesfatin-1 levels before and after the first (*t* = -0.436, *P* = 0.666, **Figure 2A**), third (*t* = 1.739, *P* = 0.093, **Figure 2B**), and sixth (*t* = 0.363, *P* = 0.720, **Figure 2C**) MECT sessions in elderly patients with TRD.

**Figure 3:**
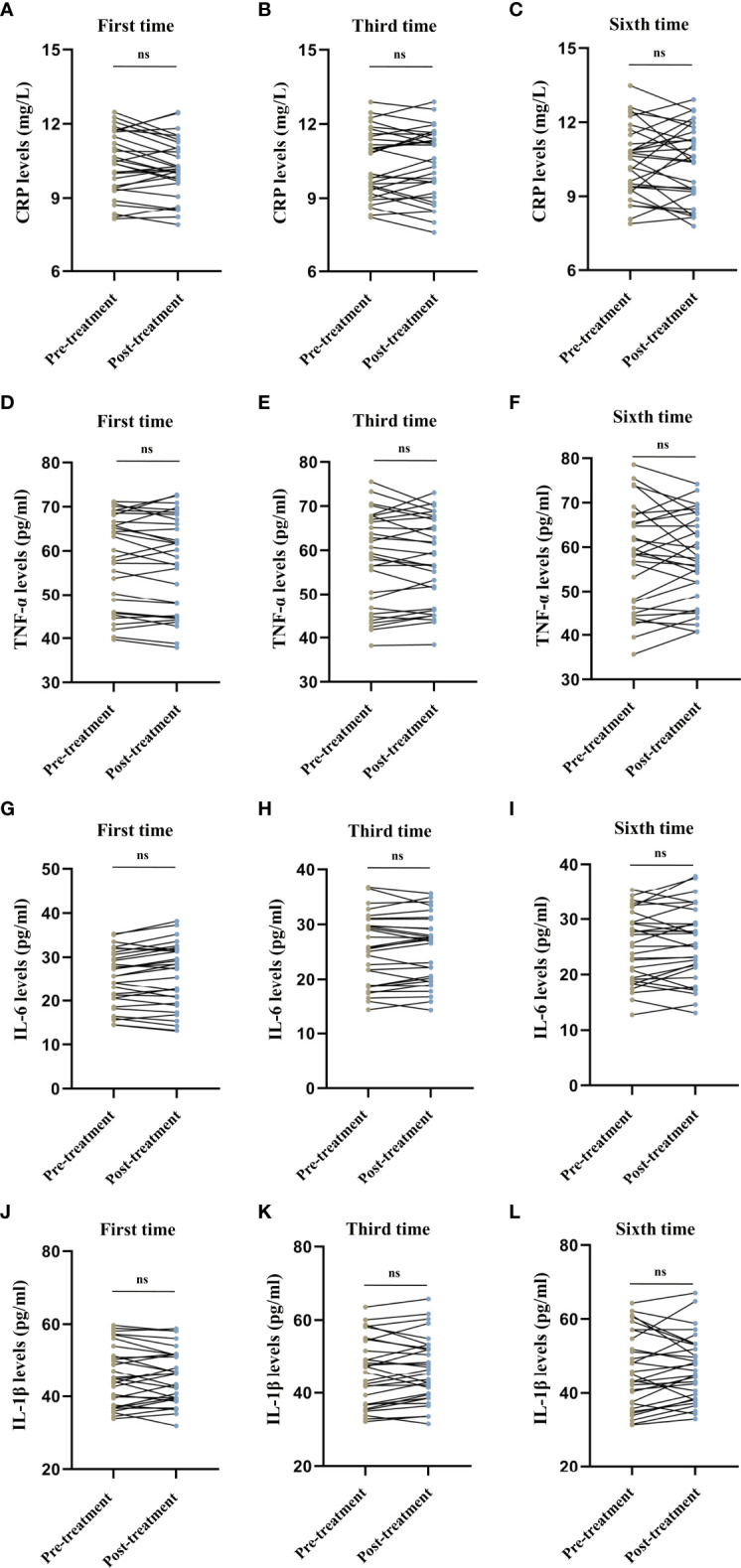
Comparison of serum C-reactive protein, tumor necrosis factor-α, interleukin-6, and interleukin-1β levels before and after modified electroconvulsive therapy. **(A–C)** Comparison of serum CRP levels before and after the first, third, and sixth MECT sessions. **(D–F)** Comparison of serum TNF-α levels before and after the first, third, and sixth MECT sessions. **(G–I)** Comparison of serum IL-6 levels before and after the first, third, and sixth MECT sessions. **(J–L)** Comparison of serum IL-1β levels before and after the first, third, and sixth MECT sessions. CRP, C-reactive protein; IL-6, interleukin-6; IL-1β, interleukin-1β; MECT; modified electroconvulsive therapy; ns, no significance; TNF-α, tumor necrosis factor-α.

We observed no difference in the serum CRP levels (first session: *t* = 1.718, *P* = 0.096, **Figure 3A**; third session: *t* = -0.073, *P* = 0.943, **Figure 3B**; sixth session: *t* = 0.517, *P* = 0.609, **Figure 3C**), TNF-α levels (first session: *t* = 1.019, *P* = 0.317, **Figure 3D**; third session: *t* = 0.443, *P* = 0.661, **Figure 3E**; sixth session: *t* = 0.087, *P* = 0.931, **Figure 3F**), IL-6 levels (first session: *t* = -1.700, *P* = 0.100, **Figure 3G**; third session: *t* = -0.308, *P* = 0.760, **Figure 3H**; sixth session: *t* = -1.100, *P* = 0.280, **Figure 3I**), and IL-1β levels (first session: *t* = 0.400, *P* = 0.692, **Figure 3J**; third session: *t* = -1.171, *P* = 0.251, **Figure 3K**; sixth session: *t* = -0.687, *P* = 0.497, **Figure 3L**) before and after MECT in elderly patients with TRD.

### Long-Term Effects of Modified Electroconvulsive Therapy on the Hamilton Depression Rating Scale-24 and Mini-Mental State Examination Scores

**Figure 4:**
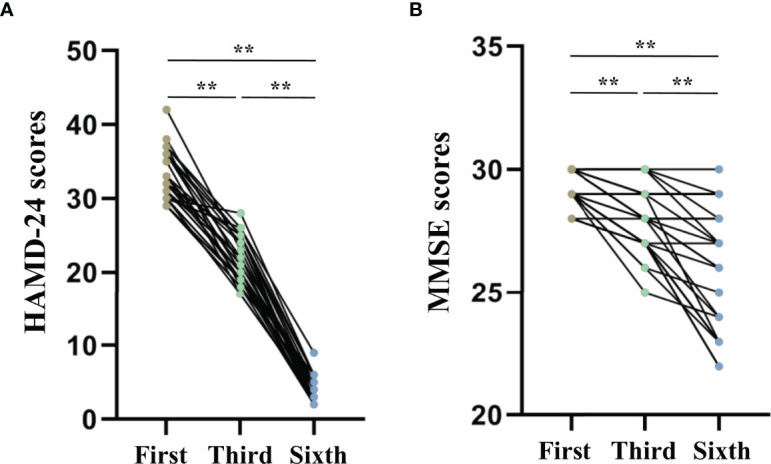
Comparison of The Hamilton Depression Rating Scale-24 and the Mini-Mental State Examinations scores across three time points (before the first, third, and sixth modified electroconvulsive therapy sessions). **(A)** Comparison of HAMD-24 scores. **(B)** Comparison of MMSE scores. **P < 0.001 was considered statistically significant. HAMD-24: The Hamilton Depression Rating Scale-24, MECT, modified electroconvulsive therapy; MMSE, Mini-Mental State Examination.

The repeated measures ANOVA test results showed that the HAMD-24 (*F* = 1012.886, *P* < 0.001, **Figure 4A**) and MMSE (*F* = 48.976, *P* < 0.001, **Figure 4B**) scores significantly decreased after MECT in elderly patients with TRD, which indicates that MECT may minimize depressive symptoms despite adverse effects on cognition.

### Long-Term Effects of Modified Electroconvulsive Therapy on the Serum Cortisol, Nesfatin-1, and Pro-Inflammatory Cytokine Levels

**Figure 5:**
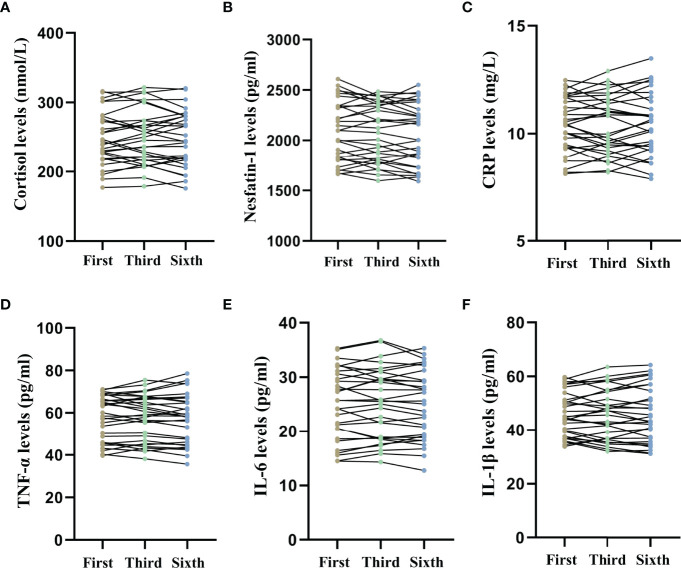
Comparison of serum cortisol, nesfatin-1, C-reactive protein, tumor necrosis factor-α, interleukin-6, and interleukin-1β levels across three time points (before the first, third, and sixth modified electroconvulsive therapy sessions). **(A)** Comparison of serum cortisol levels. **(B)** Comparison of serum nesfatin-1 levels. **(C)** Comparison of serum CRP levels. **(D)** Comparison of serum TNF-α levels. **(E)** Comparison of serum IL-6 levels. **(F)** Comparison of serum IL-1β levels. CRP: C-reactive protein, IL-6: interleukin-6, IL-1β: interleukin-1β, MECT, modified electroconvulsive therapy; TNF-α, tumor necrosis factor-α.

**Figure 5** shows the long-term effects of MECT on serum cortisol, nesfatin-1, and pro-inflammatory cytokine levels in elderly patients with TRD. The repeated measures ANOVA test results showed no significant changes in serum cortisol (*F* = 0.278, *P* = 0.676, **Figure 5A**), nesfatin-1 (*F* = 2.188, *P* = 0.121, **Figure 5B**), CRP (*F* = 0.136, *P* = 0.873, **Figure 5C**), TNF-α (*F* = 0.234, *P* = 0.704, **Figure 5D**), IL-6 (*F* = 0.507, *P* = 0.550, **Figure 5E**), and IL-1β (*F* = 0.054, *P* = 0.922, **Figure 5F**) levels across the time points used in the study (before the first, third, and sixth MECT sessions).

### Sex-Based Comparison of the Serum Cortisol, Nesfatin-1, and Pro-Inflammatory Cytokine Levels Before and After Modified Electroconvulsive Therapy

Sex-based comparison of serum indices (**Tables 1**, **2**) revealed no statistically significant differences, except for a single difference in the CRP detected at the first time point; the post-MECT serum CRP was significantly lower (*P* = 0.030) in women (**Table 2**).

**Table 1:** Comparison of the serum cortisol and nesfatin-1 levels before and after MECT treatment in different genders.

Variables	Gender	Time point	Pre-treatment	Post-treatment	*t*	*P*
Cortisol	Female (n=19)	1	246.60 ± 7.54	243.94 ± 7.35	1.207	0.243
		3	246.80 ± 7.78	252.36 ± 7.65	-1.934	0.069
		6	244.82 ± 7.98	251.89 ± 7.78	-1.532	0.143
	Male (n=11)	1	251.35 ± 14.39	248.92 ± 13.63	0.560	0.588
		3	254.41 ± 14.58	255.29 ± 16.16	-0.231	0.822
		6	252.17 ± 14.75	251.83 ± 17.26	0.049	0.962
Nesfatin-1	Female (n=19)	1	2070.81 ± 69.96	2084.75 ± 70.32	-0.711	0.486
		3	2033.91 ± 62.00	2007.39 ± 68.34	1.336	0.198
		6	2012.39 ± 69.81	2000.36 ± 67.69	0.336	0.741
	Male (n=11)	1	2144.34 ± 87.96	2138.64 ± 74.73	0.220	0.831
		3	2159.78 ± 93.09	2131.64 ± 84.37	1.066	0.311
		6	2144.70 ± 88.08	2136.83 ± 88.50	0.153	0.882

**Table 2:** Comparison of the serum CRP, TNF-α, IL-6, and IL-1β levels before and after MECT treatment in different genders.

Variables	Gender	Time point	Pre-treatment	Post-treatment	*t*	*P*
CRP	Female (n=19)	1	10.35 ± 0.29	10.07 ± 0.25	2.355	0.030
		3	10.29 ± 0.29	10.44 ± 0.33	-1.247	0.228
		6	10.34 ± 0.33	10.39 ± 0.36	-0.228	0.822
	Male (n=11)	1	10.52 ± 0.40	10.52 ± 0.41	-0.029	0.978
		3	10.58 ± 0.46	10.34 ± 0.46	1.513	0.161
		6	10.64 ± 0.45	10.29 ± 0.45	1.093	0.300
TNF-α	Female (n=19)	1	60.82 ± 2.31	59.98 ± 2.35	1.413	0.175
		3	61.01 ± 2.39	60.71 ± 2.18	0.430	0.673
		6	60.86 ± 2.65	59.48 ± 2.22	1.110	0.282
	Male (n=11)	1	52.31 ± 2.85	52.52 ± 3.31	-0.310	0.763
		3	52.06 ± 2.75	51.94 ± 2.52	0.139	0.892
		6	51.31 ± 2.87	53.47 ± 2.98	-1.755	0.110
IL-6	Female (n=19)	1	26.19 ± 1.25	26.82 ± 1.50	-1.464	0.160
		3	26.25 ± 1.36	26.40 ± 1.27	-0.376	0.711
		6	26.01 ± 1.29	26.70 ± 1.44	-1.121	0.277
	Male (n=11)	1	23.19 ± 2.33	23.58 ± 2.60	-0.822	0.430
		3	23.57 ± 2.19	23.55 ± 2.11	0.092	0.929
		6	23.17 ± 2.20	23.32 ± 2.17	-0.234	0.819
IL-1β	Female (n=19)	1	47.54 ± 1.91	47.02 ± 1.78	0.807	0.430
		3	47.63 ± 2.21	48.23 ± 2.07	-0.873	0.394
		6	48.09 ± 2.41	48.46 ± 2.09	-0.311	0.759
	Male (n=11)	1	42.62 ± 2.24	42.93 ± 2.03	-0.343	0.739
		3	42.52 ± 2.38	43.21 ± 2.43	-0.752	0.469
		6	42.14 ± 2.62	43.16 ± 2.33	-0.771	0.459

With regard to long-term effects of MECT (**Table 3**), we observed no statistically significant sex-based differences in the serum indices, except for a single difference in the serum nesfatin-1 level; the nesfatin-1 level was significantly lower (*P* = 0.041) in women at all three time points (before the first, third, and sixth MECT sessions).

**Table 3:** Comparison of the serum cortisol, nesfatin-1, CRP, TNF-α, IL-6, and IL-1β levels in different genders at three time points (before the first, third, and sixth MECT treatments) .

Variables	Gender	*F*	*P*
cortisol	Female (n=19)	0.154	0.781
	Male (n=11)	0.367	0.612
Nesfatin-1	Female (n=19)	3.493	0.041
	Male (n=11)	0.201	0.819
CRP	Female (n=19)	0.120	0.888
	Male (n=11)	0.269	0.767
TNF-α	Female (n=19)	0.027	0.930
	Male (n=11)	1.073	0.338
IL-6	Female (n=19)	0.209	0.745
	Male (n=11)	0.432	0.655
IL-1β	Female (n=19)	0.323	0.726
	Male (n=11)	0.145	0.757

### Comparison of the Serum Cortisol, Nesfatin-1, and Pro-Inflammatory Cytokine Levels Before and After Modified Electroconvulsive Therapy in the Low- and High-Cortisol Groups

Patients were categorized into low- and high-cortisol groups based on serum cortisol levels (< or > the 50^th^ percentile value of all samples). We observed no significant differences in the serum cortisol, nesfatin-1, CRP, TNF-α, IL-6, and IL-1β levels before and after the first, third, and sixth MECT sessions in the low- and high-cortisol groups (**Tables 4**, **5**).

**Table 4:** Comparison of the serum cortisol and nesfatin-1 levels before and after MECT treatment in the low-cortisol group and high-cortisol group.

Variables	Groups	Time point	Pre-treatment	Post-treatment	*t*	*P*
cortisol	Low (n=15)	1	217.11 ± 4.89	216.83 ± 5.86	0.091	0.929
		3	220.43 ± 5.55	225.07 ± 6.68	-1.587	0.135
		6	221.48 ± 7.33	221.13 ± 7.04	0.075	0.941
	High (n=15)	1	279.57 ± 6.21	274.70 ± 5.67	1.765	0.099
		3	278.75 ± 7.72	281.80 ± 8.53	-0.837	0.416
		6	273.56 ± 8.30	282.60 ± 8.38	-1.483	0.160
Nesfatin-1	Low (n=15)	1	2050.92 ± 88.32	2036.54 ± 76.56	0.685	0.505
		3	2007.66 ± 81.19	1965.75 ± 75.31	2.125	0.052
		6	1980.47 ± 81.11	1961.81 ± 72.87	0.495	0.628
	High (n=15)	1	2144.61 ± 63.95	2172.47 ± 67.29	-1.263	0.227
		3	21.52.46 ± 63.11	2140.18 ± 71.32	0.507	0.620
		6	2141.34 ± 71.27	2139.00 ± 75.83	0.052	0.959

**Table 5:** Comparison of the serum CRP, TNF-α, IL-6, and IL-1β levels before and after MECT treatment in the low-cortisol group and high-cortisol group.

Variables	Groups	Time point	Pre-treatment	Post-treatment	*t*	*P*
TNF-α	Low (n=15)	1	59.62 ± 2.43	59.24 ± 2.61	0.589	0.565
		3	59.11 ± 2.53	58.35 ± 2.24	1.008	0.331
		6	59.69 ± 3.02	58.46 ± 2.56	0.893	0.387
	High (n=15)	1	55.78 ± 2.99	55.25 ± 3.03	0.826	0.423
		3	56.35 ± 3.04	56.63 ± 2.91	-0.385	0.706
		6	55.03 ± 2.99	56.10 ± 2.67	-0.838	0.416
IL-1β	Low (n=15)	1	47.05 ± 2.22	46.42 ± 2.03	0.923	0.372
		3	47.56 ± 2.49	47.87 ± 2.62	-0.393	0.700
		6	46.50 ± 2.72	48.26 ± 2.68	-1.449	0.169
	High (n=15)	1	44.42 ± 2.04	44.63 ± 1.90	-0.257	0.801
		3	43.96 ± 2.26	44.90 ± 1.92	-1.282	0.221
		6	45.31 ± 2.60	44.78 ± 1.80	0.420	0.681
IL-6	Low (n=15)	1	24.84 ± 1.47	25.34 ± 1.73	-1.166	0.263
		3	24.96 ± 1.48	24.99 ± 1.48	-0.060	0.953
		6	24.96 ± 1.48	25.28 ± 1.60	-0.513	0.616
	High (n=15)	1	25.34 ± 1.88	25.92 ± 2.13	-1.199	0.251
		3	25.57 ± 1.88	25.72 ± 1.74	-0.397	0.697
		6	24.98 ± 1.83	25.64 ± 1.91	-1.009	0.330
CRP	Low (n=15)	1	10.47 ± 0.34	10.29 ± 0.31	1.124	0.280
		3	10.44 ± 0.37	10.44 ± 0.35	-0.048	0.963
		6	10.39 ± 0.41	10.28 ± 0.39	0.376	0.712
	High (n=15)	1	10.36 ± 0.33	10.18 ± 0.31	1.275	0.223
		3	10.35 ± 0.34	10.36 ± 0.39	-0.053	0.958
		6	10.50 ± 0.34	10.42 ± 0.41	0.343	0.737

With regard to long-term effects of MECT (**Table 6**), we observed no significant differences in serum indices, except for a single detected difference in the serum nesfatin-1 level, which was significantly decreased (*P* = 0.014) in the low-cortisol group across all time points (before the first, third, and sixth MECT sessions).

**Table 6:** Comparison of the serum cortisol, nesfatin-1, CRP, TNF-α, IL-6, and IL-1β levels at three time points (before the first, third, and sixth MECT treatments) in the low-cortisol group and high-cortisol group.

Variables	Groups	*F*	*P*
cortisol	Low	0.904	0.389
	High	1.124	0.324
Nesfatin-1	Low	5.123	0.014
	High	0.092	0.912
CRP	Low	0.110	0.897
	High	0.106	0.476
TNF-α	Low	0.259	0.658
	High	1.380	0.266
IL-6	Low	0.061	0.889
	High	0.874	0.428
IL-1β	Low	1.145	0.333
	High	1.263	0.292

**Figure 6:**
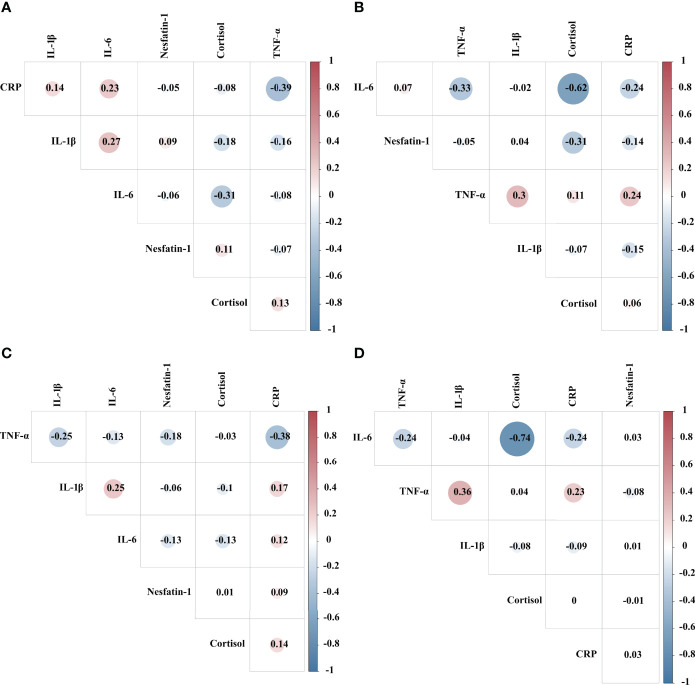
Correlation analysis showing comparison of serum cortisol, nesfatin-1, C-reactive protein, tumor necrosis factor-α, interleukin-6, and interleukin-1β levels in the low- and high-cortisol groups before and after the first modified electroconvulsive therapy session. **(A)** Correlation analysis showing comparison between serum cortisol, nesfatin-1, CRP, TNF-α, IL-6, and IL-1β levels in the low-cortisol group before the first MECT session. **(B)** Correlation analysis showing comparison between serum cortisol, nesfatin-1, CRP, TNF-α, IL-6, and IL-1β levels in the high-cortisol group before the sixth MECT session. **(C)** Correlation analysis showing comparison between serum cortisol, nesfatin-1, CRP, TNF-α, IL-6, and IL-1β levels in the low-cortisol group after the sixth MECT session. **(D)** Correlation analysis showing comparison between serum cortisol, nesfatin-1, CRP, TNF-α, IL-6, and IL-1β levels in the high-cortisol group after the sixth MECT session. CRP, C-reactive protein; IL-6, interleukin-6; IL-1β, interleukin-1β; MECT, modified electroconvulsive therapy; TNF-α, tumor necrosis factor-α.

Correlation analysis was performed using Pearson’s test to compare serum cortisol, nesfatin-1, CRP, TNF-α, IL-6, and IL-1β levels between the low- and high-cortisol groups before and after the first MECT session. Results showed that the serum cortisol level was negatively correlated with the serum IL-6 level before (*r* = -0.617, *P* = 0.014) and after the first MECT session (*r* = -0.737, *P* = 0.002) in the high-cortisol group (**Figure 6**).

### Comparison of Intergroup Differences in Serum Cortisol, Nesfatin-1, and Pro-Inflammatory Cytokine Levels Before and After The Sixth Treatment Based On Increased and Decreased Levels of Serum Indices

In view of the fact that the aforementioned serum indices increased in a few patients and decreased in others before and after the sixth treatment, patients were categorized into two groups based on the increase and decrease in serum indices before and after the sixth session.

Patients were categorized into a cortisol-increase (n = 12) and a cortisol-decrease group (n = 18) (**Table S1**). We compared serum nesfatin-1, CRP, TNF-α, IL-6, and IL-1β levels before and after the sixth treatment and observed no significant intergroup differences in the levels of these serum indices.

Similar results were observed for other serum indices, including nesfatin-1 (**Table S2**), CRP (**Table S3**), TNF-α (**Table S4**), IL-6 (**Table S5**), and IL-1β (**Table S6**).

## Discussion

This study highlights that MECT decreased the HAMD-24 and MMSE scores, and no statistically significant changes were observed in pre- and post-MECT serum cortisol, nesfatin-1, and pro-inflammatory cytokine levels. We observed no significant changes in serum cortisol, nesfatin-1, and pro-inflammatory cytokine levels across any time point (before the first, third, and sixth MECT sessions). Additionally, the serum cortisol level was negatively correlated with the serum IL-6 level before and after the first MECT session in patients with a high cortisol level (> the 50^th^ percentile value of all samples). These results suggest that MECT may be useful for management of depression and does not significantly affect serum cortisol, nesfatin-1, and pro-inflammatory cytokine levels in elderly patients with TRD.

The HAMD-24 is the most widely used scale for patient selection and follow-up in studies performed to investigate treatments for depression (21, 22). The MMSE scale, recommended by the American Academy of Neurology Guidelines, is the most common tool used to screen for cognitive impairment in clinical, research, and community settings (23). Therefore, in this study, we used the HAMD-24 and MMSE tools to evaluate depression and cognitive impairment, respectively. Consistent with the fact that MECT is effective treatment for severe major depression, the results of this study showed that MECT significantly decreased the HAMD-24 scores. Together with the results that no significant changes were observed in serum cortisol, nesfatin-1, and pro-inflammatory cytokine levels after MECT, which further suggests that MECT does not affect serum cortisol, nesfatin-1, and pro-inflammatory cytokine levels but reduces depressive symptoms in patients with TRD. Several evidence-based studies have reported that most patients with MDD who received MECT developed cognitive adverse effects (5, 24, 25). Similarly, the MMSE scores were slightly decreased in elderly patients with TRD who received MECT.

The efficacy of ECT is shown to be associated with normalization of HPA axis dysregulation (26). In contrast, several studies have reported no significant association between serum cortisol levels and ECT outcomes (27, 28). In the present study, we observed no statistically significant differences in serum cortisol levels between short- and long-term MECT in elderly patients with TRD; the basis of this conflicting association between serum cortisol levels and MECT outcomes remains unclear. Notably, this observation may be attributable to the following factors: (1) Age differences: Participants in the present study were elderly patients; however, a previous study has reported an association between cortisol levels and ECT outcomes in adults aged > 18 years (29). (2) Differences in samples measured: Evaluation of hair or salivary cortisol provides useful information regarding the recent history of an individual’s HPA function; therefore, some studies have investigated the association between the HPA axis and ECT based on measurement of hair and salivary cortisol levels (30–32). In our study, we measured serum cortisol levels. (3) Differences in disease severity and subtypes: Increased cortisol levels are observed in patients who were known to experience their first episode of MDD but not in patients with recurrent MDD (33). The lack of an association between serum cortisol levels and MECT outcomes may be attributable to the unique composition of our patient sample; we predominantly included elderly patients with TRD in the present study.

Recent research has reported the role of nesfatin-1, a newly identified potent anorexigenic 82-amino acid peptide, in regulation of emotional processes, with emphasis on the functional implications of depression (34). Reportedly, patients with MDD show elevated nesfatin-1 levels in the peripheral blood (35). Moreover, a positive correlation was observed between serum nesfatin-1 levels and the severity of depression (10, 11) and a negative correlation between suicidal ideation scores and nesfatin-1 levels (36), which supports the role of nesfatin-1 in the pathogenesis of depression. To our knowledge, only one study has investigated the effects of ECT on serum nesfatin-1 levels in men with manic episodes (12). The present study is the first to investigate the effects of MECT on circulating serum nesfatin-1 levels in elderly patients with TRD. Our results indicate that short- and long-term MECT did not significantly affect serum nesfatin-1 levels, which supports the fact that serum nesfatin-1 levels are not associated with MECT outcomes in elderly patients with TRD.

Emerging evidence has indicated an association between inflammation and depression in older patients (37). Recent studies corroborate that higher baseline inflammation is associated with a poor response to pharmacological antidepressant therapies (38). Studied have reported conflicting findings regarding the effects of ECT on serum pro-inflammatory cytokine levels. Many studies have observed an anti-inflammatory effect of ECT (39–41); however, a few others have reported no post-ECT changes in inflammatory markers (42–44). In the present study, we observed no significant post-MECT effects on serum pro-inflammatory cytokine levels in elderly patients with TRD. Currently, the basis of these contradictions remains unclear; therefore, further large-scale studies are warranted to confirm these findings, and *in vitro* and *in vivo* studies should be performed to establish the possible mechanisms underlying these findings, as well as to gain a deeper understanding of the mechanism of action of MECT in patients with different stages and subtypes of depression and to identify patients who can benefit from MECT.

Interestingly, we observed a negative correlation between serum IL-6 and cortisol levels in patients with high cortisol levels. The exact mechanisms underlying this negative correlation remain unclear; however, the following contributors may play a role: (1) Age was positively correlated with inflammatory markers, including serum IL-6, and serum cortisol was negatively correlated with age (45). The present study included only elderly patients. Therefore, the correlation between serum cortisol and IL-6 levels may be attributable to age. (2) Cortisol is the strongest endogenous anti-inflammatory hormone (46); therefore, patients with relatively high cortisol levels may show lower inflammation levels.

Following are the limitations of this study: (a) The single-center small-scale design of this study is a drawback, particularly because sex-based subgroup categorization further reduced the cohort size, which may have resulted in lack of some expected differences (for example, in inflammatory cytokine levels) in this study. (b) We could not control the effects of adverse lifestyle factors including smoking, obesity, exercise, and sleep, which are known to affect immune function. (c) Antidepressant drug administration was not discontinued prior to MECT, which may have affected the serum cortisol, nesfatin-1, and pro-inflammatory cytokine levels.

In conclusion, this study is the first to highlight that MECT did not significantly affect serum cortisol, nesfatin-1, and pro-inflammatory cytokine levels in elderly patients with TRD. Multicentric large-scale studies are warranted in future to validate these findings.

## Data Availability Statement

The original contributions presented in the study are included in the article/**Supplementary Material**. Further inquiries can be directed to the corresponding author.

## Ethics Statement

The studies involving human participants were reviewed and approved by Anhui Mental Health Center. The patients/participants provided their written informed consent to participate in this study.

## Author Contributions

BD, XW, QX, XZ, and XX contributed to conception and design of the study. XW, BD, FY, YC, and YX organized the database. BD, FY, and YX performed the statistical analysis. BD and YX wrote the first draft of the manuscript. All authors contributed to manuscript revision, read, and approved the submitted version.

## Funding

This project was funded by the Research Fund Project of Hefei Fourth People’s Hospital (Project No: 2019009) and the Research Fund Project of Anhui Medical University (Project No: 2019xkj203).

## Conflict of Interest

The authors declare that the research was conducted in the absence of any commercial or financial relationships that could be construed as a potential conflict of interest.

## Publisher’s Note

All claims expressed in this article are solely those of the authors and do not necessarily represent those of their affiliated organizations, or those of the publisher, the editors and the reviewers. Any product that may be evaluated in this article, or claim that may be made by its manufacturer, is not guaranteed or endorsed by the publisher.
