# Supplementary material for: Effects of Modified Electroconvulsive Therapy on Serum Cortisol, Nesfatin-1, and Pro-inflammatory Cytokine Levels in Elderly Patients With Treatment-Resistant Depression
Source: Front Endocrinol (Lausanne). 2022 Jun 16;13:904005. doi: 10.3389/fendo.2022.904005 (PMC9243445; doi:10.3389/fendo.2022.904005)
Supplement: Supplementary file 1 [file Table_1.docx]

**Table S1 Comparison of demographic data and the difference of serum cortisol, nesfatin-1, CRP, TNF-α, IL-6, and IL-1β levels before and after the sixth treatment in the cortisol-increase group and the cortisol-decrease group**

| Variables | Cortisol-increase | Cortisol-decrease | *t/* | *P* |
| --- | --- | --- | --- | --- |
| Age | 66.50±2.29 | 66.33±1.80 | 0.058 | 0.954 |
| Gender (female/male) | 8/4 | 11/7 | 0.096 | 0.757 |
| BMI | 23.24±1.21 | 22.88±0.88 | 0.243 | 0.810 |
| HAMD | 33.75±1.11 | 32.28±0.54 | 1.196 | 0.249 |
| Cortisol | 17.22±3.10 | -18.73±2.83 | 8.376 | <0.001 |
| Nesfatin-1 | 54.61±46.37 | -18.90±36.48 | 1.256 | 0.220 |
| CRP | -0.17±0.34 | 0.27±0.21 | -1.162 | 0.255 |
| TNF-α | 0.65±1.18 | -0.29±1.39 | 0.477 | 0.637 |
| IL-6 | -0.78±0.55 | -0.30±0.66 | -0.511 | 0.613 |
| IL-1β | -0.70±1.41 | -0.55±1.18 | -0.085 | 0.933 |

**Table S2 Comparison of demographic data and the difference of serum cortisol, nesfatin-1, CRP, TNF-α, IL-6, and IL-1β levels before and after the sixth treatment in the nesfatin-1-increase group and the nesfatin-1-decrease group**

| Variables | Nesfatin-1-increase | Nesfatin-1-decrease | *t* | *P* |
| --- | --- | --- | --- | --- |
| Age | 68.36±1.87 | 64.69±1.98 | 1.333 | 0.193 |
| Gender (female/male) | 9/5 | 10/6 | 0.010 | 0.919 |
| BMI | 22.54±1.16 | 23.45±0.87 | -0.643 | 0.525 |
| HAMD | 32.79±0.70 | 32.94±0.85 | -0.135 | 0.894 |
| Cortisol | 0.40±4.24 | -8.51±6.17 | 1.156 | 0.257 |
| Nesfatin-1 | 156.08±24.85 | -116.88±15.96 | 9.241 | <0.001 |
| CRP | 0.32±0.24 | -0.10±0.28 | 1.101 | 0.280 |
| TNF-α | 0.64±1.32 | -0.40±1.38 | 0.542 | 0.592 |
| IL-6 | -0.78±0.67 | -0.24±0.61 | -0.591 | 0.559 |
| IL-1β | -0.34±1.02 | -0.85±1.44 | 0.284 | 0.779 |

**Table S3 Comparison of demographic data and the difference of serum cortisol, nesfatin-1, CRP, TNF-α, IL-6, and IL-1β levels before and after the sixth treatment in the CRP-increase group and the CRP-decrease group**

| Variables | CRP-increase | CRP-decrease | *t* | *P* |
| --- | --- | --- | --- | --- |
| Age | 67.33±1.96 | 65.00±1.88 | 0.817 | 0.421 |
| Gender (female/male) | 11/6 | 8/5 | 0.032 | 0.858 |
| BMI | 22.83±0.99 | 23.32±1.00 | -0.340 | 0.736 |
| HAMD | 32.56±0.76 | 33.33±0.80 | -0.683 | 0.500 |
| Cortisol | -5.73±4.10 | -2.27±7.67 | -0.432 | 0.669 |
| Nesfatin-1 | 25.92±38.61 | -12.62±44.64 | 0.645 | 0.524 |
| CRP | 0.76±0.15 | -0.90±0.18 | 7.195 | <0.001 |
| TNF-α | 0.20±1.37 | -0.09±1.26 | 0.147 | 0.884 |
| IL-6 | -1.07±0.64 | 0.37±0.50 | -1.618 | 0.117 |
| IL-1β | -1.04±1.07 | 0.03±1.57 | -0.585 | 0.563 |

**Table S4 Comparison of demographic data and the difference of serum cortisol, nesfatin-1, CRP, TNF-α, IL-6, and IL-1β levels before and after the sixth treatment in the TNF-α-increase group and the TNF-α-decrease group**

| Variables | TNF-α-increase | TNF-α-decrease | *t* | *P* |
| --- | --- | --- | --- | --- |
| Age | 67.40±1.90 | 65.40±2.07 | 0.712 | 0.482 |
| Gender (female/male) | 7/8 | 12/3 | 3.589 | 0.058 |
| BMI | 23.72±1.06 | 22.33±0.92 | 0.982 | 0.335 |
| HAMD | 33.80±0.88 | 31.93±0.61 | 1.749 | 0.091 |
| Cortisol | 0.51±5.91 | -9.21±4.87 | 1.270 | 0.215 |
| Nesfatin-1 | 23.89±39.39 | -2.89±43.57 | 0.456 | 0.652 |
| CRP | 0.00±0.28 | 0.20±0.25 | -0.529 | 0.601 |
| TNF-α | 4.32±0.90 | -4.16±0.60 | 7.828 | <0.001 |
| IL-6 | 0.13±0.65 | -1.12±0.59 | 1.419 | 0.167 |
| IL-1β | -0.46±1.02 | -0.76±1.49 | 0.162 | 0.872 |

**Table S5 Comparison of demographic data and the difference of serum cortisol, nesfatin-1, CRP, TNF-α, IL-6, and IL-1β levels before and after the sixth treatment in the IL-6-increase group and the IL-6-decrease group**

| Variables | IL-6-increase | IL-6-decrease | *t* | *P* |
| --- | --- | --- | --- | --- |
| Age | 63.80±1.19 | 67.70±1.96 | -1.339 | 0.191 |
| Gender (female/male) | 7/3 | 12/8 | 0.287 | 0.592 |
| BMI | 25.31±0.91 | 21.88±0.86 | 2.489 | 0.019 |
| HAMD | 33.70±0.86 | 32.45±0.71 | 1.069 | 0.294 |
| Cortisol | -10.09±7.68 | -1.48±4.36 | -1.051 | 0.302 |
| Nesfatin-1 | 3.65±49.51 | 13.93±36.58 | -0.165 | 0.871 |
| CRP | 0.23±0.36 | 0.03±0.22 | 0.485 | 0.631 |
| TNF-α | 1.75±1.36 | -0.75±1.23 | 1.250 | 0.221 |
| IL-6 | 2.26±0.35 | -1.87±0.36 | 7.229 | <0.001 |
| IL-1β | 0.13±1.48 | -0.98±1.12 | 0.586 | 0.563 |

**Table S6 Comparison of demographic data and the difference of serum cortisol, nesfatin-1, CRP, TNF-α, IL-6, and IL-1β levels before and after the sixth treatment in the IL-1β-increase group and the IL-1β-decrease group**

| Variables | IL-1β-increase | IL-1β-decrease | *t* | *P* |
| --- | --- | --- | --- | --- |
| Age | 64.09±2.36 | 67.74±1.69 | -1.276 | 0.212 |
| Gender (female/male) | 8/3 | 11/8 | 0.660 | 0.417 |
| BMI | 23.04±0.91 | 23.02±0.95 | 0.010 | 0.992 |
| HAMD | 33.45±1.15 | 32.53±0.57 | 0.805 | 0.428 |
| Cortisol | 3.08±7.64 | -8.65±4.06 | 1.491 | 0.147 |
| Nesfatin-1 | 11.01±44.71 | 10.21±38.62 | 0.013 | 0.990 |
| CRP | 0.19±0.33 | 0.04±0.23 | 0.392 | 0.698 |
| TNF-α | 0.77±1.51 | -0.32±1.24 | 0.547 | 0.589 |
| IL-6 | 0.65±0.64 | -1.15±0.56 | 2.040 | 0.051 |
| IL-1β | 4.69±0.86 | -3.68±0.58 | 8.313 | <0.001 |
